# Sudden deterioration of renal function in a patient with nephrotic syndrome and a very high hepatitis B viral DNA load

**DOI:** 10.12861/jrip.2012.14

**Published:** 2012-01-01

**Authors:** Hamid Nasri, Muhammed Mubarak

**Affiliations:** ^1^Department of Nephrology, Division of Nephropathology, Isfahan University of Medical Sciences, Isfahan, Iran; ^2^Department of Histopathology, Sindh Institute of Urology and Transplantation (SIUT), Karachi, Pakistan

**Keywords:** Hepatitis B virus, Renal failure, Glomerulonephritis

**Implication for health policy/practice/research/medical education:**

In this case, the clinical history and morphologic lesions of kidney biopsy of a 38 year-old Afghan origin male are discussed. The patient presented with nephrotic syndrome and positive hepatitis B surface antigen (HBsAg). Renal biopsy was mostly consistent with membranoproliferative glomerulonephritis (MPGN) type I. Two months after prednisolone therapy, patient’s condition suddenly deteriorated and acute renal failure was found. The patient underwent dialysis. During evaluation, >2×10^7^ IU/ml of viral DNA of hepatitis B was found. In the second biopsy, crescentic glomerulonephritis was evident. After adding lamivudine to the regimen, serum creatinine decreased and stabilized at 1 mg/dl. Patient was discharged in stable condition and the lamivudine was continued

## Case presentation

**Figure 1:**
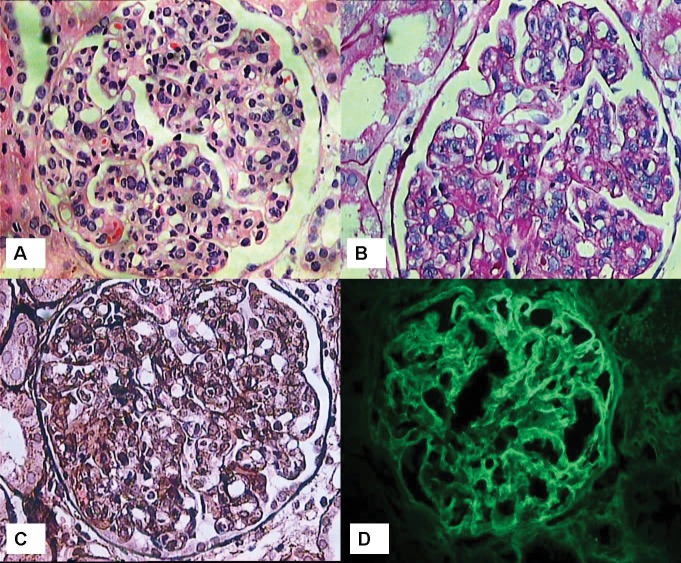
The morphological and immunofluorescence (IF) findings on first renal biopsy. A) High-power view showing diffuse mesangial proliferation with lobulation and prominent interpositioning of expanded mesangium into peripheral capillary loops. There is also some neutrophilic exudation. Capillary lumena are reduced in diameter and only a few are patent. (Hematoxylin and Eosin (H&E, ×400). B) Another glomerulus showing diffuse mesangial proliferation and interpositioning of expanded mesangium into peripheral capillary loops with marked reduction of capillary lumena. Adjacent tubules show degenerative changes with loss of brush border and luminal dilatation. (Period acid-Schiff stain, ×400). C) Silver stain highlighting the same morphological features as seen in sections A and B. (Jones methenamine silver stain, ×400). D) Diffuse, thick band-like positivity of IgG along peripheral capillary walls and to a minor degree in the mesangium also. (IF, IgG, ×400).

**Figure 2:**
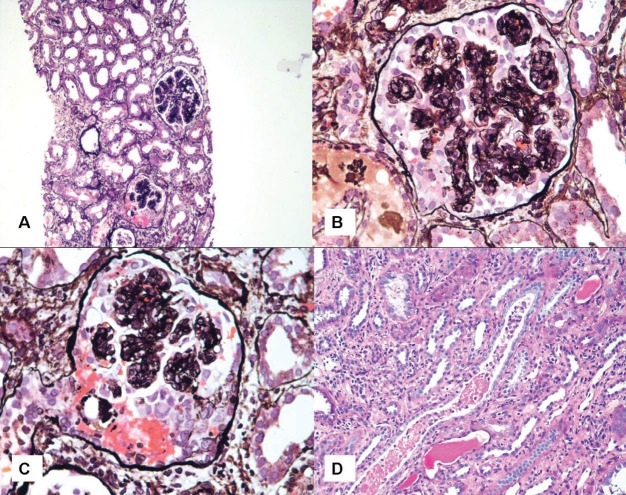
The morphological findings on second renal biopsy. A) Low-power view showing one glomerulus with diffuse mesangial proliferation with prominent lobulation. Another glomerulus in the lower field shows cellular crescent formation superimposed on background changes of membranoproliferative GN (MPGN). (Jones methenamine silver stain, ×100). B) Medium-power view showing one glomerulus with early cellular proliferation in Bowman’s space (early crescent formation) on the background changes of MPGN. (Jones methenamine silver stain, ×200). C) Another glomerulus showing florid cellular crescent formation. (Jones methenamine silver stain, ×200). D) Medium-power view showing hyaline casts and necrotic debris in some tubular lumena. There is also moderate interstitial inflammation in the interstitium. (Period acid-Schiff stain, ×200).

A 38-year-old Afghan origin male presented with generalized edema. He gave the history of intravenous (IV) drug abuse. Edema started 2 weeks before admission. Urine sediment had dysmorphic red blood cells (RBCs) and serum creatinine was 1 mg/dl. The amount of proteinuria was 4560 mg/day. Secondary evaluation revealed a negative human immunodeficiency virus (HIV) and hepatitis C virus (HCV) tests. HBsAg was positive. Erythrocyte sedimentation rate (ESR) was 25 mm/1st hr. Liver functions tests were within normal limits. C reactive protein (CRP) was negative. Cardiac doppler echography was normal. Kidney sonography showed right and left kidneys of 11.6×5 and 12.2×4.6 cm^2^ in dimensions, respectively. Other secondary evaluation tests including serologic activity of systemic lupus erythematosus (SLE), cryoglobulins, complement levels, antineutrophilic cytoplasmic antibody (ANCA), anti-phospholipid antibody panel tests and serum protein electrophoresis were within normal range. For further evaluation, a kidney biopsy was performed. On renal biopsy, 13 glomeruli were included, none of them was sclerotic. All glomeruli displayed mesangial proliferation accompanied by endocapillary proliferation in 1/3 of the glomeruli. The glomeruli had a lobulated pattern. On silver staining, spikes were absent, however, glomerular basement membrane (GBM) was thickened. Extracapillary proliferation was absent. Interstitial inflammation, fibrosis and tubular atrophy were absent, however, tubular cells showed significant degenerative changes and RBC casts were observable. On immunofluorescence (IF) study of 9 obtained glomeruli, linear band-like intra-capillary wall deposits of IgG were observed (3+ on a scale of 0 to 3+ intensity). Also, there was 1+ of C3 deposition, while C1q, IgA and IgM were not found. There was also 1+ positivity of IgG in the mesangial area. Granular deposits of IgG along the GBM were absent. The morphologic lesions and the IF study was mostly consistent with membranoproliferative glomerulonephritis (MPGN), type I. The patient was treated with 50 mg/day prednisolone. Two months after starting the treatment and during the third periodic check up, patient complained of urinary frequency and dysuria. Primary evaluation revealed a serum creatinine of 0.9 mg/dl and a bacteriuria in urine analysis. Clarithromycin (500 mg/twice day) was added to the regimen. One week after treatment, patient was referred to the emergency ward due to confusion and malaise. Except for the previously mentioned drugs (prednisolone and clarithromycin), there was no history of other drug intake. Also, there was no history of gastro-enteritis. Primary evaluation showed a serum creatinine of 20 mg/dl. A jugular catheter was installed and hemodialysis was started. Previous treatment with 50 mg/day of prednisolone was also continued. New evaluation of biochemical parameters also showed negative HIV and HCV serologic tests. On real-time polymerase chain reaction (PCR) >2×107 IU/ml of viral DNA of hepatitis B was found. At this time lamivudine was added to the patient’s regimen. Other serologic tests including ANCA, SLE panel test, anti-phospholipid antibodies, anti-GBM antibody and C3, C4 and CH50 levels were within normal limits. Liver function tests and coagulation panel were disturbed. General condition of the patient improved after seven hemodialysis sessions. At this stage, there was no more need for dialysis and serum creatinine gradually decreased. At this time, we decided to perform a second kidney biopsy. After correcting the coagulation profile using fresh frozen plasma, the second renal biopsy was performed. On the second kidney biopsy, of 12 obtained glomeruli, 10 showed crescents and none of the glomeruli was sclerotic. Crescents were mostly circumferential and cellular. There was peri-glomerular infiltration, too. Destruction of Bowman’s capsule in some of the glomeruli was observed. Interstitial area harbored moderate inflammatory cell infiltration. Tubular casts, either RBCs or degenerated debris casts were numerous. Tubular dilatation was evident. Tubular cells showed marked degenerative changes. Vessels had normal morphology and architecture. On IF study, the findings were similar to those of the first biopsy. The interpretation was a proliferative glomerulonephritis involved endocapillary and extracapillary regions, mostly consistent with MPGN, Type I. Finally, serum creatinine returned to 1 mg/dl after two months of admission and the patient was discharged and returned to his country in stable condition and the lamivudine was continued (Figures 1  and 2).

## Discussion

In this case, we describe the history of a patient who had a very high viral load of HBV DNA. This case firstly presented with nephrotic syndrome and a MPGN. We could not pinpoint exactly the factor responsible for sudden aggravation of renal function and deterioration of morphologic lesions toward crescent formation. However, it is clear that adding lamivudine to the treatment improved renal functions and alleviated the need for dialysis.

HBV infection has a cosmopolitan distribution, with a high prevalence in most developing countries (1). Chronic infection with the HBV is associated with various immunopathological manifestations, involving autoantibody production and immune-complex-related pathologies (1-3). In kidney, it is generally accepted that persistent viral infections lead to immune complex-mediated nephropathy and may explain the HBV-associated nephropathy (HBVAN). Various mechanisms have been suggested to explain the damage to renal tissue by HBV; a) deposition of immune complexes containing viral antigens and host antibodies, b) direct cytopathic effects of viral infection and finally, c) virus-induced specific immunological mechanisms damaging the kidney and the adverse effects of virus-induced cytokines or mediators in kidney tissue. However, the most probable mechanism of HBVAN is the deposition of immune complexes consisting of viral antigens and host antibodies. Various HBV antigens have been described to be deposited to the glomeruli consisting of HBsAg, HBcAg, and HBeAg. However, hepatitis B envelope antigen (HBeAg) in association with IgG has a central role in the pathogenesis of HBVAN (2-5). In fact, it has been found that HBeAg is the primary antigen related to the subepithelial deposits in patients with HBVAN (2-5). HBV infection may present with various forms of glomerular disease including membranous nephropathy, minimal change disease, focal segmental glomerulosclerosis, IgA nephropathy, MPGN, and mesangial proliferative glomerulonephritis (3-5).

In this patient, the interesting finding was the improvement of renal function by the addition of lamivudine to the therapeutic regimen. We conclude that lamivudine, could regress the crescents. However, it should also be noted that, performing the second biopsy and arriving at a correct diagnosis was also one of the most important measures for the treatment of this patient.

## Authors’ contributions

MM and HN wrote the manuscript equally.

## Conflict of interests

The author declared no competing interests.

## Ethical considerations

Ethical issues (including plagiarism, misconduct, data fabrication, falsification, double publication or submission, redundancy) have been completely observed by the author.

## Financial /Support

None.
